# Autologous Fat Grafting in Severe Lower Extremity Asymmetries: Report of Four Cases

**DOI:** 10.7759/cureus.402

**Published:** 2015-12-13

**Authors:** Juan Monreal

**Affiliations:** 1 Plastic Surgery, Hospital Moncloa

**Keywords:** fat grafting, human adipose tissue, lower extremity reconstruction, stromal vascular fraction, mesenchymal stem/stromal cells, reconstruction

## Abstract

Background: Lower extremity asymmetries are challenging problems in plastic and aesthetic surgery practice. Regardless of their origin, atrophies and asymmetries can be extremely varied and difficult to solve with simple techniques.

Objectives:  The author reports his experience in the treatment of four patients suffering from severe lower extremity atrophy and asymmetry of different etiologies with autologous fat grafting.

Methods: A total of four cases are presented. Patient selection was based on the severity of atrophy and asymmetry. Two patients were treated with two sessions of simple fat grafting and two patients with one session of cell-enriched fat grafting. The end point in each session was determined by tension/blanching of soft tissues. All patients were followed up for at least 12 months after the last session. During the postoperative follow-up, variables, such as objective volume improvement, objective girth loss, return to daily activities, and patient satisfaction, were analyzed.

Results: The initial analysis of postoperative results showed a good patient satisfaction rate with no relevant complications and an early return to daily activities. Estimated mean volume improvement for simple fat grafting cases was estimated as 44% after two treatments. Mean volume improvement in cell-enriched fat grafting cases was estimated as 25% after only one treatment.

Conclusions: Autologous fat grafting is a safe, effective, and reliable technique to perform aesthetic and reconstructive reshaping of a lower extremity in cases of atrophy or severe asymmetry. Depending on the preoperative soft tissue compliance, cell-assisted fat grafting will play an important role in reducing the number of sessions to perform.

## Introduction

Reconstruction of the lower extremities is one of the broadest and most diverse disciplines in plastic surgery. The origin of the problems, suitable to immediate or delayed reconstruction, is extremely broad, from complex trauma or oncologic surgery to degenerative, iatrogenic, or birth defects. Many of these patients require multiple surgeries to obtain adequate functional results, allowing them to develop daily activities as normal as possible. One of the main goals of treatment in most patients is to provide a good quality of soft tissue coverage that will ensure an adequate quality of life and long-term durability to the reconstruction.

There can be significant differences in the type of limb atrophy - asymmetry after each type of surgery or pathology. A complex trauma that has needed one or more flaps will end up with quite different sequels than a pure muscle atrophy caused by neuropathy. However, many of these patients have several factors in common: noticeable asymmetries between both limbs, different degrees and distribution of soft tissue compliance, and atrophy. These circumstances, coupled with the specific vascular anatomy of the lower limb, pose major constraints to treatment selection. 

Traditionally, most surgical treatments have sought to improve symmetry and/or contour to the lower extremity with the use of implants or flaps [1-4]. These approaches can improve the contours or volume, but it could have a great impact on the quality of the already damaged tissues. Implant/flap-based reconstructions are aggressive surgical techniques often rejected by some patients due to a new scar in addition to previous surgical episodes.

The author reports his experience in lower extremity reconstruction with fat grafting. Based in a clinical experience of 12 patients, four of them with the highest degrees of atrophy and asymmetry were selected.

## Materials and methods

**Figure 1:**
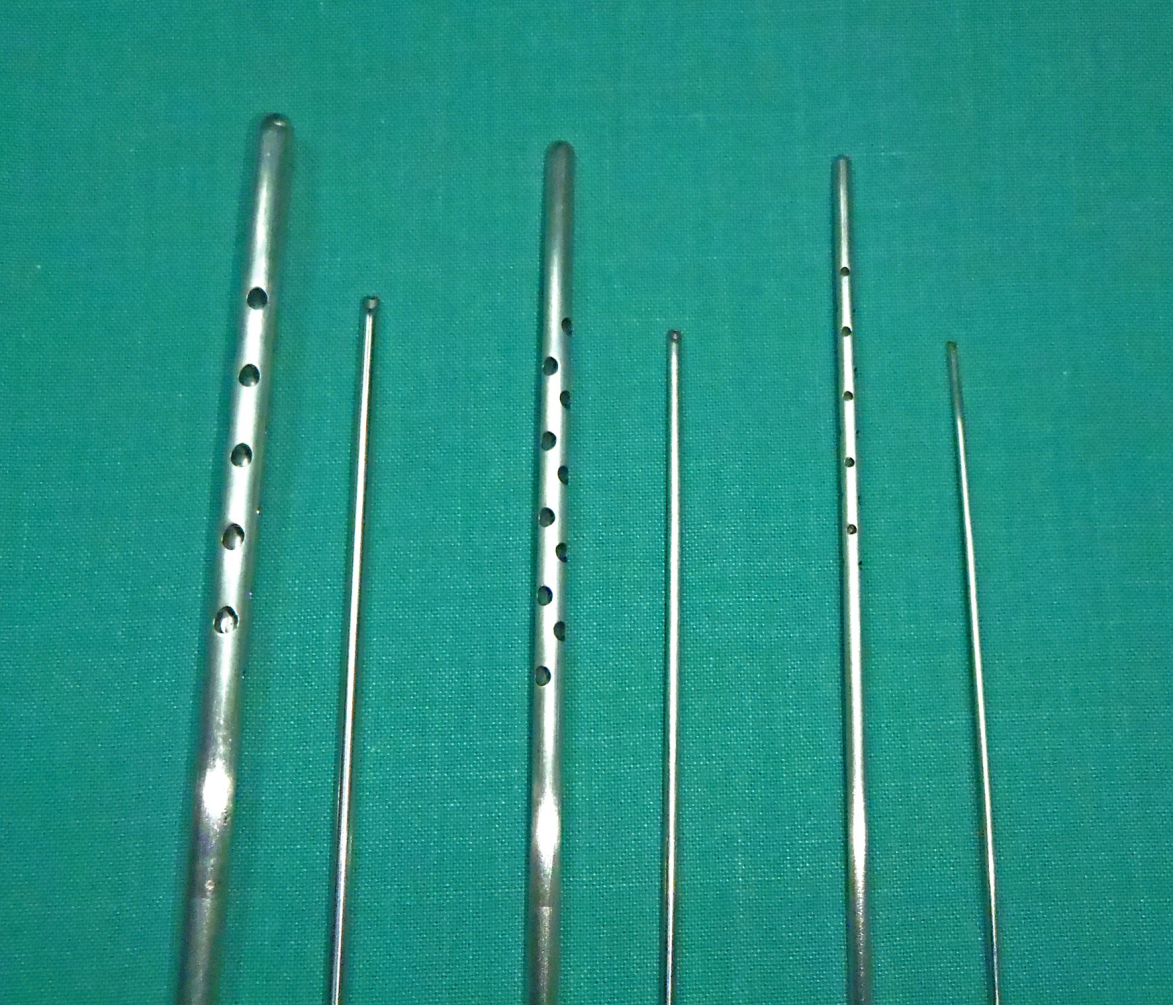
Collecting and injecting cannulas used by the author.

From 2006 to 2014, the author has used autologous fat grafting to treat a total of 12 patients with lower extremity asymmetries. Of them, four cases with severe asymmetries of different etiologies are presented. The basic criteria for selection were the severity of atrophy and the extent of involvement. The four patients presented include only those which had circumferential involvement of the calf and distal third of the leg. All patients refused to have implant/flap-based reconstructions and, indeed, their common motivation was to seek a minimally invasive technique. All of them were informed about the principles and objectives of fat grafting techniques and the need for at least two sessions in order to overcome the lack of tissue compliance and avoid fatal complications. After informed written consent, all surgeries were performed under spinal anesthesia on an outpatient basis. The donor sites selected were the abdomen, flanks, and inner thighs, changing the donor site with each new session. Fat grafting sessions were performed using the same protocols as previously described by the author [5-7]. Fat was harvested using 3 - 4 mm multi-holed cannulas and processed by washing with Ringer's lactate solution and decanting (Figure 1). Two patients were treated with cell-enriched fat grafting using the following protocol: One hundred and fifty ml of fat tissue were collected and processed to obtain the stromal vascular fraction component. The adipose tissue was rinsed with Ringer's lactate and digested with a solution containing 0.075% collagenase type IA (Worthington, Lakewood, NJ, USA). The digested tissue was centrifuged, supernatant fluid was removed after centrifugation, and the pellet was then resuspended in Ringer Lactate solution. The distribution of fat grafts was performed using 1.4 to 2 mm blunt tipped cannulas from at least eight access points and extending from subfascial to hypodermic planes. Whenever possible, the fat was evenly distributed circumferentially to allow for tissue recruitment in an attempt to increase girth. The end limit of the total grafted volume was imposed by soft tissue compliance, stopping infiltration at the first sign of skin whitening or tension. Postoperative care consisted of antibiotic prophylaxis during four days and the use of pressure garments for two weeks in the donor site and for eight weeks in the recipient site begining its use at the end of surgery. All patients were allowed to return to their normal activities on the sixth postoperative day and were encouraged not to perform any sports or outdoor activities for at least two months.

### Case presentation

Case 1 is a 33-year-old female patient who had multiple surgeries performed during childhood to treat a congenital malrotation affecting both knees and both ankles. The result was satisfactory in that a normalized standing and ambulation was achieved. The aesthetic sequelae consisted of the presence of longitudinal scars in the anterior surface of both knees extending to the distal third of anterior thigh and proximal third of the pretibial area. The patient showed a marked asymmetry in contour and volume of both legs. The soft tissue coverage was rated as normal but showed a significant lack of compliance in her right leg. Magnetic resonance imaging revealed no significant vascular abnormality but confirmed the extreme atrophy and asymmetry in muscle bellies of both legs. The patient was informed about the need to perform at least two sessions in order to expand soft tissues without having untoward results.

**Figure 2:**
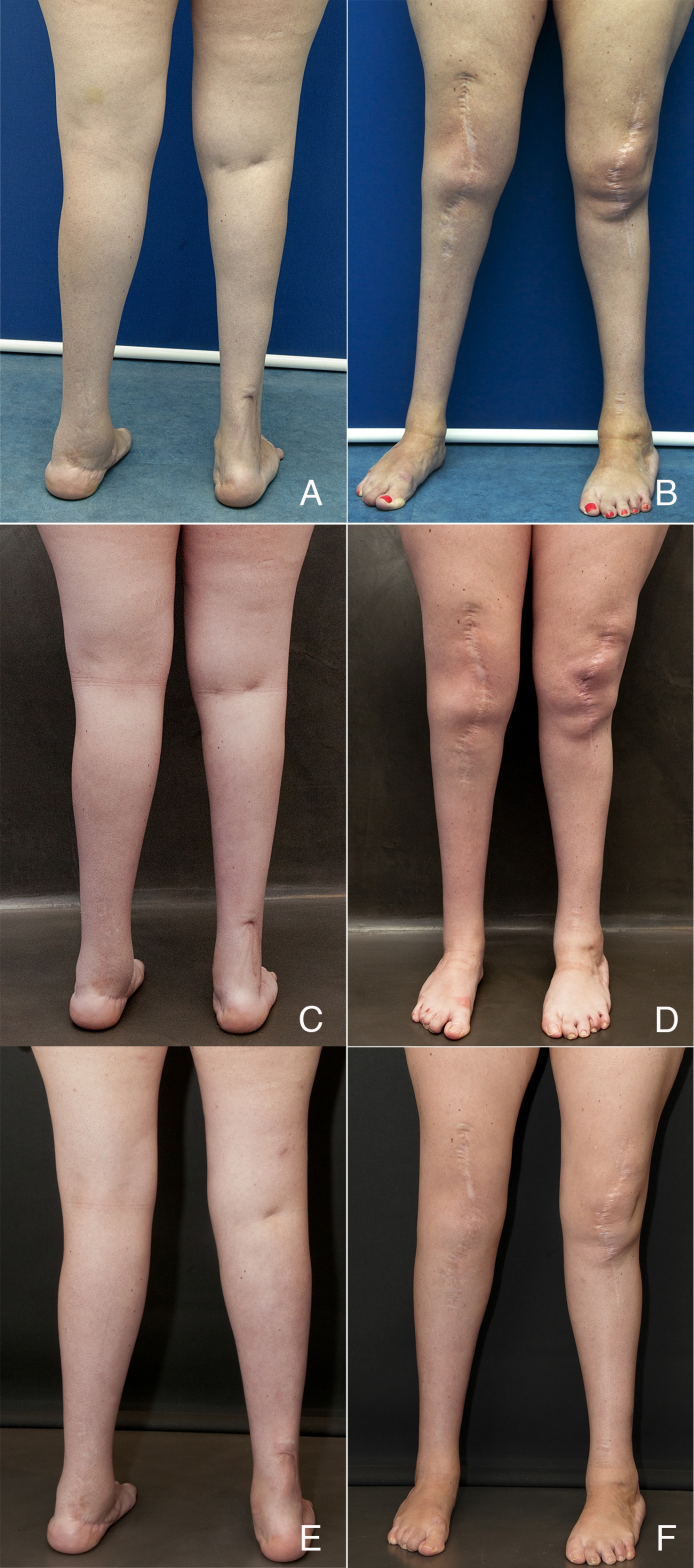
Case 1 (A, B) Preoperative view of Case 1; (C, D) Postoperative views 6 months after first session; (E, F) 16 months after second session. Courtesy of Cirugía Plástica Ibero-Latinoamericana.

During the first session, 270 cc of adipose tissue was grafted in her right leg, 170 cc to in left leg, and 20 cc were distributed under and inside the scars. The distribution of injected fat covered the whole girth in her right leg and was more selective in her left leg. The scars were grafted at different levels in order to obtain a better shape and texture. There was no intramuscular infiltration at any level. Six months later, a second session was performed in which 320 cc of fat were grafted to the right leg, 170 cc to the left, and 20 cc were used to treat scars. Pre- and postoperative images show a marked and stable improvement in contour, volume, symmetry, and scar quality after 16 months (Figure 2). Girth improved from 28 cm preoperatively to 35 cm in her right leg and from 30 to 35 cm in her left leg. These measures represent a 53% increase in volume in the right leg and 34% in the left leg.

**Figure 3:**
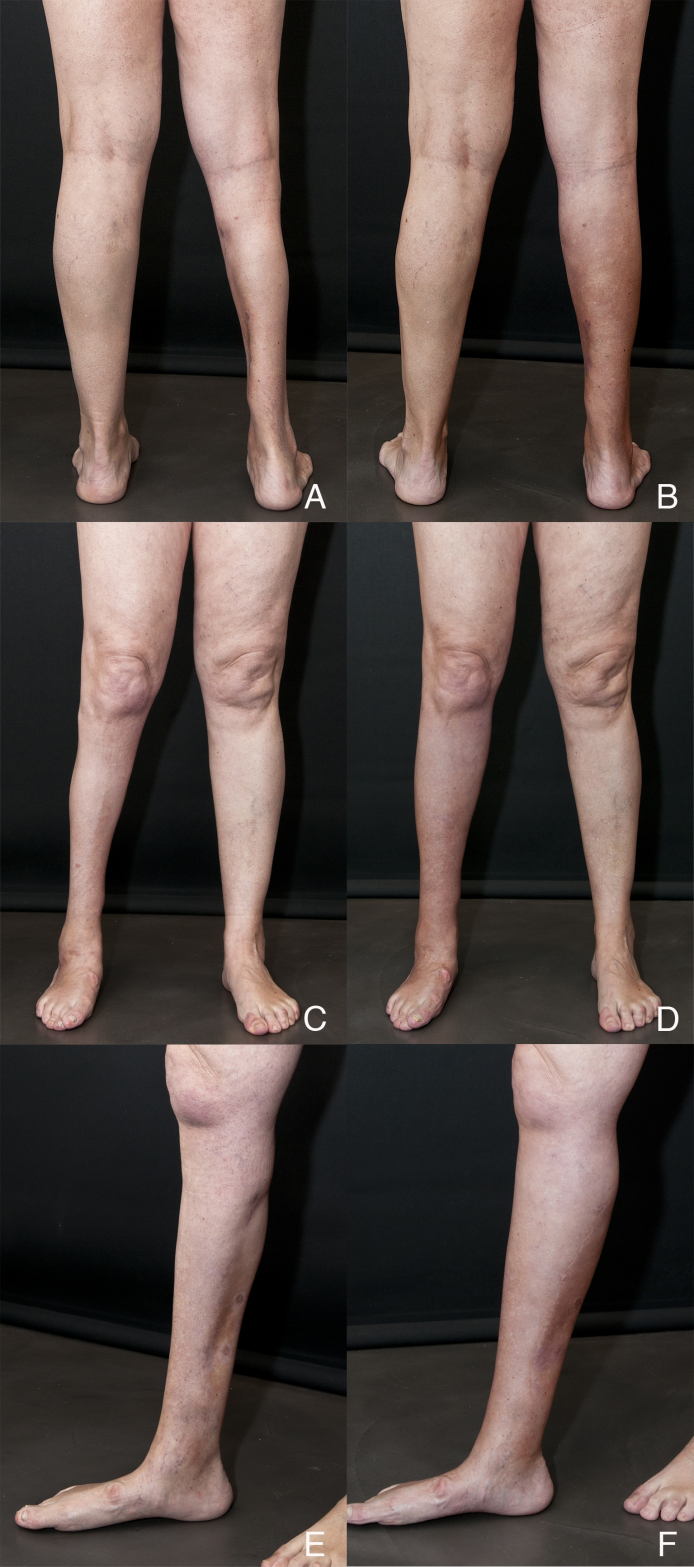
Case 2 (A, C, E) Preoperative view of Case 2; (B, D, F) Postoperative views 12 months after second session. Courtesy of Cirugía Plástica Ibero-Latinoamericana.

Case 2 is a 50-year-old female patient that was referred to our center in order to improve the symmetry of her legs after explantation of a calf prosthesis implanted 25 years before by another surgeon. The implant had caused a marked impairment of the surrounding tissues due to a long-term rupture. As a result, there was an extensive involvement with cutaneous fibrosis and medial gastrocnemius atrophy that involved the whole length of the inner right leg. MRI scan confirmed physical findings and no vascular abnormalities or compromise. The first session consisted of grafting of 250 cc of adipose tissue, which was distributed all around calf area and inner distal third. It was necessary to judiciously use conventional sharp needles to adequately free some extremely adherent areas of fibrosis. Four months later, a second session was performed using the same fat grafting volume and distribution. Figure 3 shows the pre- and postoperative images of the patient one year after the second session. Adherent zones are still visible in the lower inner third of the leg, but improvements in volume and contour are stable and, most importantly, the quality of skin coverage significantly improved from the patient's point of view. Girth improved from 26 cm preoperatively to 31.5 cm after the second session. This represents a 45% increase in volume after second treatment.

**Figure 4:**
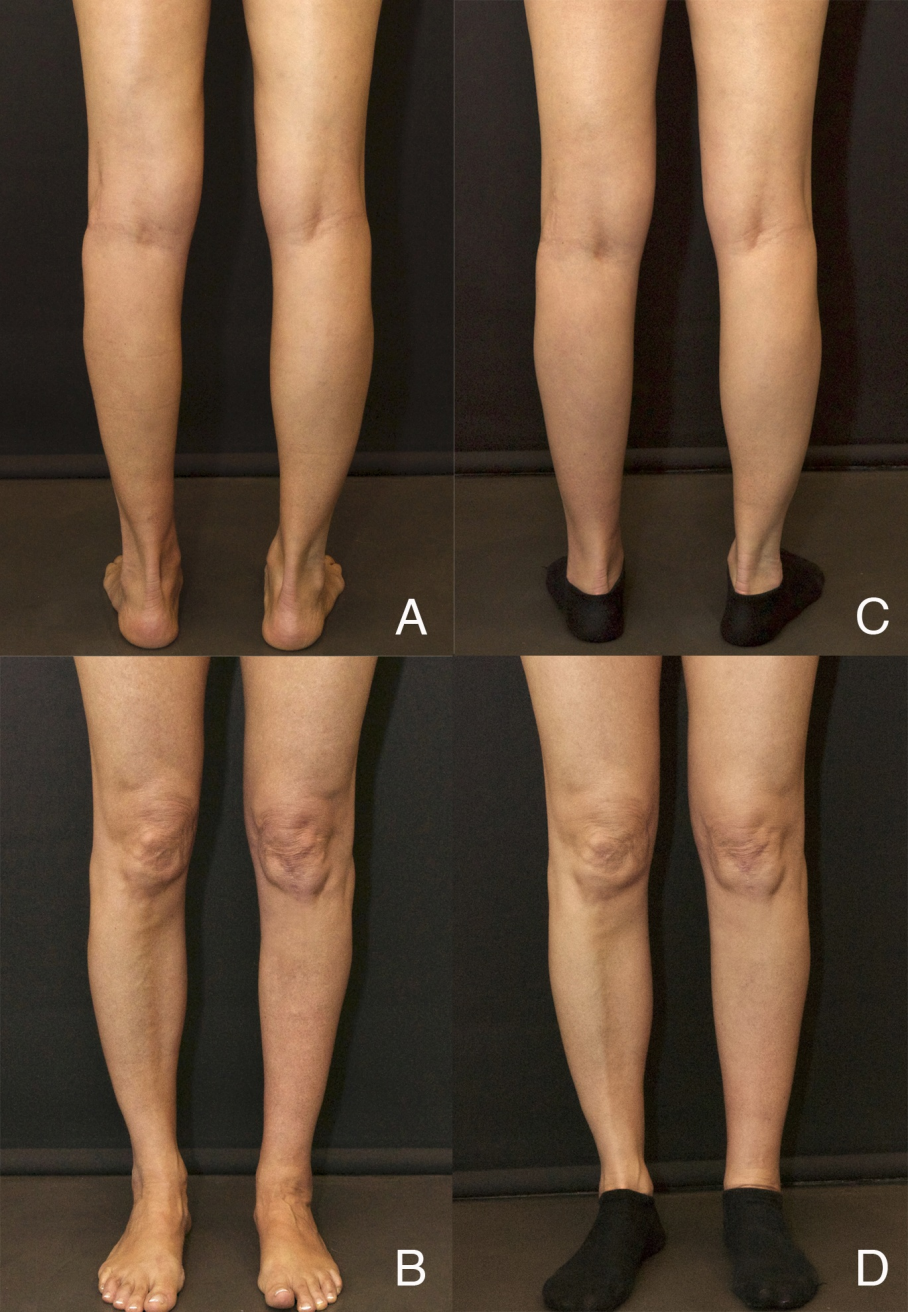
Case 3 (A, B) Preoperative views of Case 3; (C, D) Postoperative views 15 months after single cell-enriched fat grafting session.

Case 3 is a 48-year-old female patient that presented with isolated medial and lateral muscle atrophy in her left calf due to iatrogenic neuropathy. Soft tissue thickness and compliance were considered good so a single session of cell-assisted fat grafting was planned in an attempt to get a one-step improvement. The treatment consisted of injecting 130 cc of fat enriched with stromal vascular fraction (SVF) cells for a total concentration of 8.25x10^5^ viable cells/ml of injected fat. Figure 4 shows the preoperative and postoperative result 15 months after the last session, showing a stable improvement in volume and contour. Girth improved from 29.0 cm preoperatively to 31.0 cm compared to 31.5 cm on the healthy side. This represents a 12% increase in volume after only one treatment.

**Figure 5:**
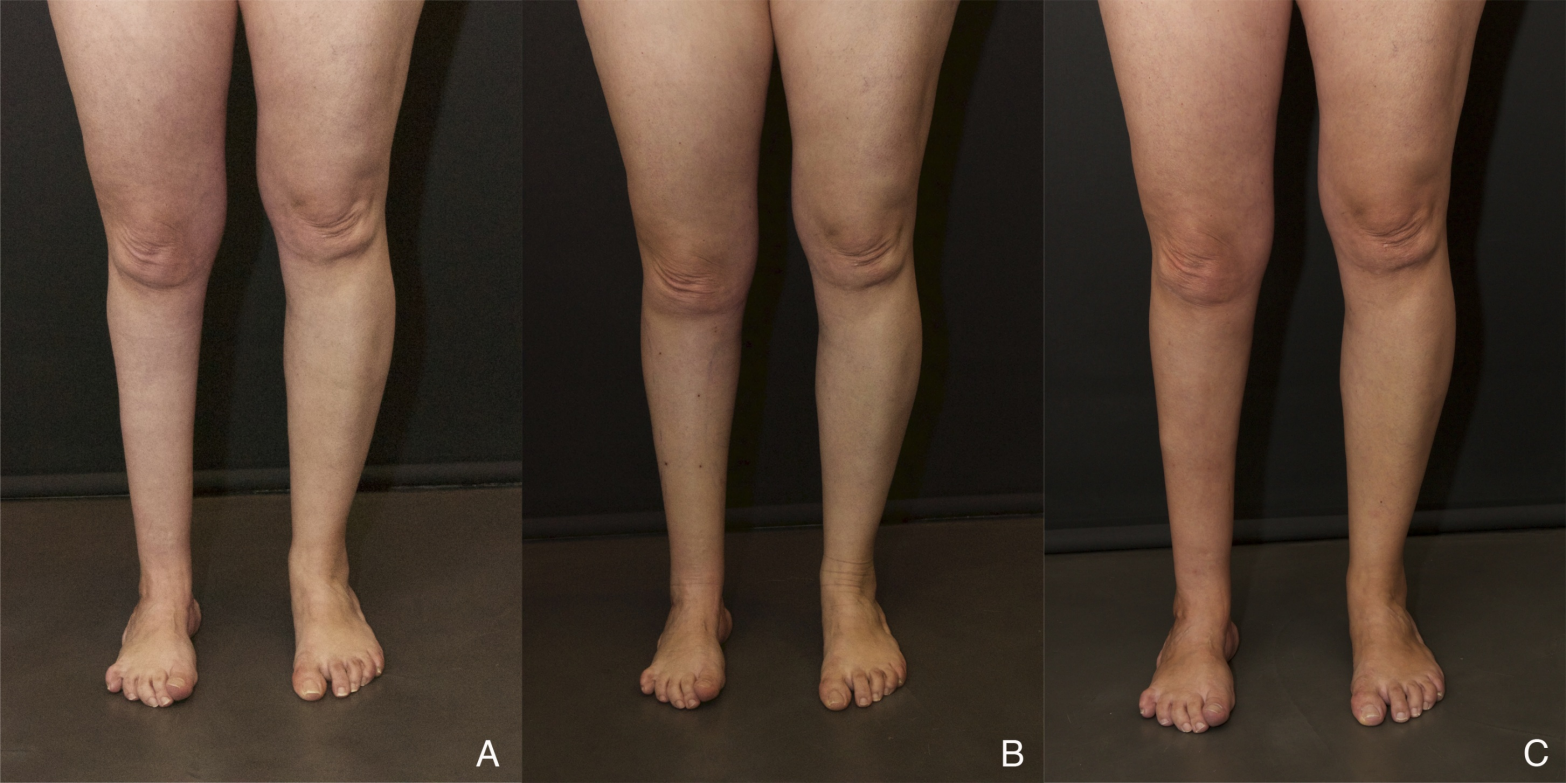
Case 4 (A) Preoperative views of Case 4; (B) Postoperative views 15 days and (C) 12 months after a single cell-enriched fat grafting session.

Case 4 is a 45-year-old female patient with post-polio calf muscle atrophy and multiple scars derived from ankle arthrodesis. Physical examination revealed good soft tissue coverage, but there was a considerable lack of compliance. The patient was informed about the need to perform at least two sessions to achieve some degree of symmetry. During the first session, a total of 230 cc of fat were placed circumferentially in her right leg in order to improve girth. Injected fat was enriched with stromal vascular fraction (SVF) cells to a final concentration of 7.32x10^5^ viable cells/ml of fat. Figure 5 shows the preoperative and postoperative result 12 months after the first session, showing good but modest improvement in volume and contour, even with the use of cell-assisted fat grafting. Girth improved from 27.5 cm preoperatively to 32.5 cm compared to 37 cm on the healthy side. These numbers represent a volume augmentation of 39% after only one treatment.

## Results

When performing volume augmentation of a cylindrical area, it is relatively easy to follow up the improvement by using a measuring tape and a reference point. Table 1 shows preoperative and postoperative data for each case. For every patient at each visit, the same reference point on the calf was marked, which served as the location for serial height and girth measurements in the treated zone. The author could then calculate the volume of the zone using the cylinder volume formula $V = Bh = \pi r^2 h$. These volumes were used to calculate the percentage of increment in each patient. Patients were followed up each week during the first month and every two months thereafter. Calf girth taken at the reference point was measured at each visit, but these values were only referenced to the measure obtained at the second postoperative week to dismiss the increase in volume resulting from postoperative edema and tumescent fluid contained in fat grafts. From the fourth postoperative month, the author did not observe any significant decreases in measurements. The preoperative volume increased 12% in Case 3 and 39% in Case 4 after one treatment in cell-enriched cases, whereas it increased 45% in Case 2, 53% in Case 1, right leg, and 34% in Case 1, left leg after two treatments in non-cell-assisted cases. One of the patients suffered from some prolonged ankle edema caused by an outdoor activity that resolved with rest and pressotherapy. Subjective factors (patient driven), such as symmetry, texture, and quality of tissue and scars, significantly improved. All the patients rated the postoperative course as good.

**Table 1:** Treatment protocol and data analysis for each case Using girth measurements at a reference point, the height of treated area a preoperative, and postoperative volume estimation was calculated using the cylinder volume formula V=Bh=πr2h

	Case 1	Case 2	Case 3	Case 4
Nº of Treatments	2	2	1	1
Fat Grafting Protocol	No Cell-Enrichment	No Cell-Enrichment	Cell-Enrichment	Cell-Enrichment
First Treatment (cc)	270 cc right leg	250 cc	130 cc (8,25x105 cells/ml of fat)	230 cc (7,32x105 cells/ml of fat)
	170 cc left leg			
Second Treatment (cc)	320 cc right leg	250 cc	-	-
	170 cc left leg			
Left Ø Preop (cm)	30,0	32,5	29,0	37,0
Left Ø Postop (cm)	35,0	32,5	31,0	37,0
Right Ø Preop (cm)	28,0	26,0	31,5	27,5
Right Ø Postop (cm)	35,0	31,5	31,5	32,5
Left Volume Pre. (cc)	1.591	1.867	1.507	2.404
Left Volume Post (cc)	2.143	1.867	1.334	2.404
Right Volume Pre. (cc)	1.398	1.184	1.57	1.319
Right Volume Pst (cc)	2.143	1.727	1.57	1.846
Volume Increment	Left 34%	Left 0%	Left 12%	Left 0%
	Right 53%	Right 45 %	Right 0%	Right 39 %

## Discussion

While one might assume that fat grafting is a safe and reliable surgical technique for treating volume defects with an undeniable regenerative potential [8], reports are scarce in the literature that address the use of fat grafting in aesthetic lower extremity contouring [9-11]. It is still lower than the number of reports on the use of fat grafting in the reconstruction of post-traumatic, congenital, or iatrogenic lower leg sequela. Mojallal, et al. analyzed a series of 20 cases of traumatic, congenital, and iatrogenic origin treated with fat grafting with excellent results [12]. The use of flaps and prostheses can improve certain defects but lacks fundamental aspects, which are desirable in some of these patients: low invasiveness and regenerative potential. Additionally, flap/implant-based reconstructions create new scars or increment the length of existing ones. Injecting other soft tissue fillers, such as hyaluronic acid, may be a useful alternative, which is easy to perform with a quick recovery time, but still lacks proven biological effects and involves lifetime treatments. The use of other non-resorbable synthetic fillers in soft tissues with compromised microvascular networks can create additional complications, such as infection or extrusion. Patients who already have undergone multiple interventions will not accept new complex reconstructions that have prolonged postoperative periods, includes prostheses, or adds more scars. 

Fat grafting can be a judicious and valuable alternative for these patients as it combines aesthetic improvements with tissue regeneration and long-lasting results. Properly done, it has a very low complication rate and markedly improves the microvascular network. However, it is necessary to consider several aspects that may influence the results and the absence of complications. When dealing with a low compliance area, it will be essential to plan a two or even three stage reconstruction in order to achieve serial tissue expansion. Therefore, it is extremely important not to overcorrect in order to avoid risks, such as massive graft necrosis or compartment syndrome. Vascularization of the distal portions of the lower limb is normally quite sensitive and can be very fragile in the presence of postsurgical or posttraumatic changes. Although the use of small diameter blunt cannulas greatly reduces the possibility of intravascular injection, it is particularly important to remember the vascular anatomy of the lower leg to take appropriate precautions to avoid any complications. These precautions must be extreme when, due to the quality of tissues or scars, the use of cannulas with a cutting bevel is mandatory.

Over the past twenty years, autologous fat grafting has emerged as a popular strategy to manage soft tissue deficits throughout the body [13]. To minimize fat graft loss, studies have shown benefits offered in using less traumatic methods of harvesting, processing, and injecting as well as using cell enrichment with adipose-derived stem cells (ASCs) contained in the stromal vascular fraction of fat tissue. In 2007, Rigotti and colleagues [14] tested the therapeutic potential of adipose-derived adults stem cells in a clinical pilot study focused on the treatment of irradiation-induced lesions. Matsumoto, et al. introduced the concept of cell-assisted lipograft (CAL), in which fat grafts are enriched with stromal vascular fractions containing ASCs isolated from adipose tissue [15]. In animal studies, subcutaneous implantation of enriched fat grafts into immunodeficient mice demonstrated 35% increased volume retention when compared to conventional fat grafting. In 2008, Yoshimura, et al. presented a preliminary study of 40 patients who underwent cell-enriched fat grafting for cosmetic breast augmentation with the findings demonstrating its safety and effectiveness compared to traditional fat grafting [16]. Although the use of enriched fat grafts has proved to be useful improving retention and local conditions of damaged tissues, some cases have limiting factors, such as tissue compliance and the degree of tissue expansion needed to accomplish results. When the recipient zone presents limited compliance and needs significant expansion, this expansion must be conducted gradually in several steps in order to avoid serious complications, such as compartment syndrome. On the other hand, in recipient sites with adequate compliance where expansion need not be serially performed, enriched fat grafting can offer the advantages of improving long-term survival and retention volume, thus reducing the number of treatments. Cases 3 and 4 are good examples of cell-enriched fat grafting with and without this limiting factor. Although fat graft retention can be considered as very good in both cases, Case 3 needed only one session to improve symmetry due to good tissue compliance, whereas in Case 4, a modest improvement in symmetry was obtained after a single session. 

Traditionally, fat grafting procedures have been performed without the use of cell enhancement with different kind of results arising from multiple variables. Although cell enhancement can be performed in every fat grafting procedure, the evidence does not support the routine use of ASCs-enriched fat grafts and the author has been able to verify this fact during his last four years of experience. The increase in operative time, expenses, and efficiency must drive its use to selected cases [17]. In the author's experience, cell-assisted fat grafting can be considered an indication whenever graft/host interface is defective, compromising cell survival, or to enhance their regenerative potential in damaged tissues by fibrosis or adhesions. However, the author has found no benefit using cell enhancement in reducing the final number of treatments in cases in which the lower extremity had low tissue compliance. This limiting factor in a delicate vascular area is only surmountable by serial expansions.

## Conclusions

Choosing the most appropriate treatment in cases of aesthetic reconstruction of the lower limbs must go through a thorough analysis of the features present in each patient. Asymmetries, their location, quantity and quality of soft tissue coverage, and the presence or absence of scars are critical factors. Although there are more treatment options available to resolve these cases, fat grafting meets several important advantages that make it a first choice technique: less aggressive, efficiency in volume improvement, and regenerative potential that benefits damaged tissues. Its main disadvantage is probably the need to perform several treatments in order to expand soft tissue coverage and overcome tissue tightness. The stability of long-term results does not seem to differ from other anatomical areas, although further experience is needed to track improvements in the longer term due to aging or weight changes.
